# Protection of ZIKV infection-induced neuropathy by abrogation of acute antiviral response in human neural progenitors

**DOI:** 10.1038/s41418-019-0324-7

**Published:** 2019-04-05

**Authors:** Ling Liu, Zhenyu Chen, Xin Zhang, Shun Li, Yi Hui, Hexi Feng, Yanhua Du, Guohua Jin, Xiaohui Zhou, Xiaoqing Zhang

**Affiliations:** 1 Brain and Spinal Cord Innovative Research Center, Tongji Hospital, Tongji University School of Medicine, Shanghai, 200065 China; 2 Key Laboratory of Reconstruction and Regeneration of Spine and Spinal Cord Injury, Ministry of Education, Shanghai, 200065 China; 3 Key Laboratory of Neuroregeneration of Shanghai Universities, Tongji University, School of Medicine, Shanghai, 200092 China; 4 Department of Anatomy and Neurobiology, The Jiangsu Key Laboratory of Neuroregeneration, Nantong University, Nantong, Jiangsu 226001 China; 5 Shanghai Public Health Clinical Center, Fudan University, Shanghai, 201508 China; 6 Tsingtao Advanced Research Institute, Tongji University, Shanghai, 200092 China; 7 Shanghai Institute of Stem Cell Research and Clinical Translation, Shanghai, 200120 China; 8 Translational Medical Center for Stem Cell Therapy, Shanghai East Hospital, Tongji University School of Medicine, Shanghai, 200120 China

**Keywords:** Neuroscience, Cell biology

## Abstract

It remains largely unknown how Zika virus (ZIKV) infection causes severe microcephaly in human newborns. We examined an Asian lineage ZIKV, SZ01, which similarly infected and demonstrated comparable growth arrest and apoptotic pathological changes in human neuroprogenitors (NPCs) from forebrain dorsal, forebrain ventral as well as hindbrain and spinal cord brain organoids derived from human pluripotent stem cells. Transcriptome profiling showed common overactivated antiviral response in all regional NPCs upon ZIKV infection. ZIKV infection directly activated a subset of IFN-stimulated genes (ISGs) in human NPCs, which depended on the presence of IRF3 and NF-κB rather than IFN production and secretion, highlighting a key role of IFN-independent acute antiviral pathway underlying ZIKV infection-caused neuropathy. Our findings therefore reveal that overactivated antiviral response is detrimental rather than protective in human NPCs, and the IFN-independent acute antiviral pathway may serve as a potential target to ameliorate ZIKV infection-triggered neuropathy.

## Introduction

Zika virus (ZIKV), an enveloped, single-stranded RNA virus of the flavivirus family, has re-emerged and spread throughout many countries recently [1]. Microcephaly is the most devastating birth defect associated with ZIKV-infected newborns. Epidemiological statistics reported a 20-fold increase in the number of newborn microcephaly cases during the outbreak of ZIKV in Brazil in 2015 [2]. Clinical case-control studies have also established solid connections between maternal ZIKV infection in the first trimester of pregnancy and microcephaly [2, 3]. The neurotropism of ZIKV and the microcephaly phenotype caused by ZIKV infection have also been demonstrated in mouse models [4–8] and recently in rhesus monkeys [9].

The innate immune system defends the host from viral infections through eliciting type I interferons (IFNs), which exert autocrine or paracrine functions to induce hundreds of IFN-stimulated genes (ISGs) [10–12]. Infection of RNA viruses or their production of RNA intermediates can also directly activate a subset of ISGs independent of type I IFN signaling through intrinsic expression or activation of IFN-regulatory factor 3 (IRF3) or nuclear factor-κB signaling pathway (NF-κB) [12–14]. The induction of ISGs through both IFN-dependent and -independent pathways is thought to be the key for host innate immunity against viruses [14].

Though mouse models can partially resemble microcephaly caused by ZIKV infection in the fetal human brain, a human-based system is of broad interest to study the pathological processes underpinning ZIKV infection. Human embryonic stem cells (hESCs) and human induced pluripotent stem cells (hiPSCs) can be efficiently specified into neural progenitor cells (NPCs), which closely mimics human embryonic neural development [15–19]. Infection with ZIKV in these pluripotent stem cell-derived human NPCs causes severe growth arrest and cell death by apoptosis and autophagy, effectively mirroring the neurotropism of ZIKV and characteristic pathological microcephaly [8, 20–23].

It is currently unknown whether ZIKV infects human NPCs with different regional identities equally or if bias exists. How ZIKV causes cell growth arrest and apoptosis in human NPCs also remains elusive. Here, we show that an Asian lineage, ZIKV strain SZ01, isolated from a patient infected in Samoa in early 2016 [24], efficiently infects the human forebrain dorsal (FD), forebrain ventral (FV), as well as hindbrain and spinal cord (H&S) NPCs derived from hESCs. The ZIKV infection leads to reduced cell viability and proliferation rate, elevated apoptosis, and triggers robust ISG activation. Unexpectedly, the acutely activated ISGs is extremely detrimental for the normal proliferation of human NPCs, which might account for the microcephaly pathology upon ZIKV infection.

## Results

### ZIKV efficiently infects hESC-derived NPCs of different regional identities

**Fig. 1:**
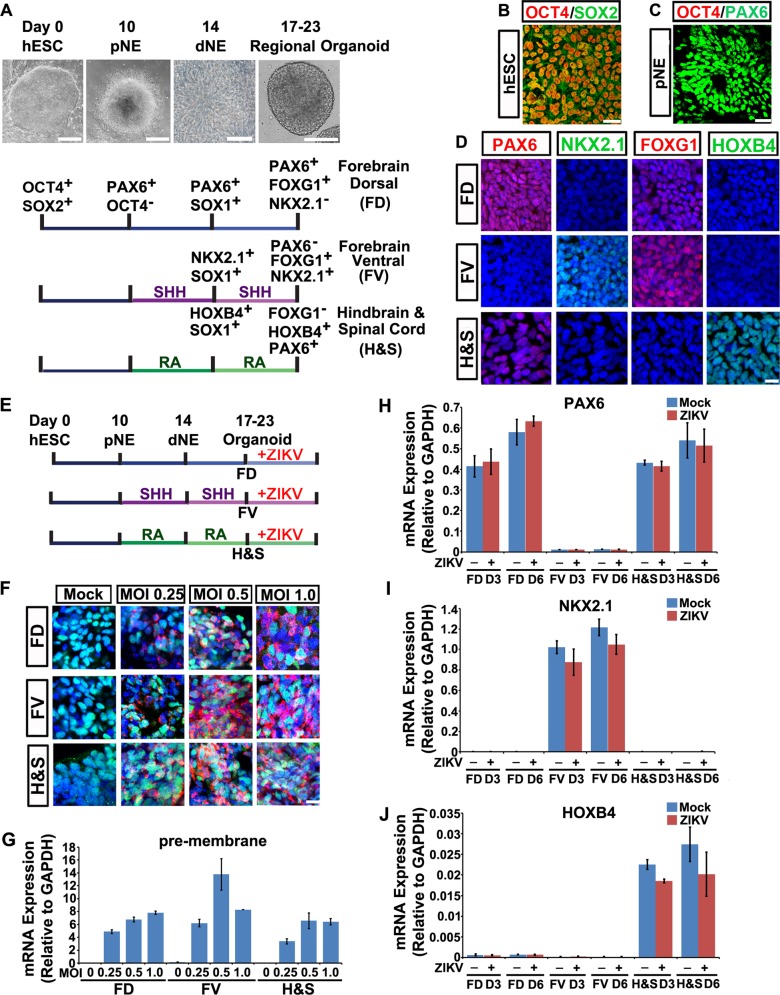
ZIKV efficiently infects hESC-derived brain organoids of different regional identities. **a** Schematic representation of hESCs patterning to regional brain organoids in vitro. Without patterning morphogens, human ESCs differentiated into forebrain dorsal (FD) NPCs at day 17. When Shh was applied from day 10 to day 17, hESCs were patterned into forebrain ventral (FV) NPCs. To generate hindbrain & spinal cord (H&S) NPCs, retinoid acid (RA) was added from day 10 to day 17. Light images show typical hESC clone at D0, columnar pNE at D10 (Scale bar, 200 μm), organized neurotube-like rosette of dNE at D14 (Scale bar, 25 μm) and regional organoid at D20 (Scale bar, 100 μm). **b** Confocal image showing undifferentiated hESCs were Oct4^+^/Sox2^+^. Scale bar, 50 μm. **c** Confocal image showing day 10 pNE cells were Pax6^+^/Oct4^−^. Scale bar, 50 μm. **d** FD, FV and H&S organoids were dissociated and plated onto coverslips for immunostaining. FD NPCs were Pax6^+^/Nkx2.1^−^/Foxg1^+^. FV NPCs were Pax6^−^/Nkx2.1^+^/Foxg1^+^. H&S NPCs were Foxg1^−^/Hoxb4^+^/Pax6^+^. Scale bar, 50 μm. **e** Schematic representation of ZIKV infection of FD, FV and H&S organoids from day 17 to day 23. **f** Confocal images stained for viral envelope proteins (red), Sox2 (green) and nuclei (blue) of ZIKV-infected FD, FV, H&S NPCs at different viral MOI (0.25, 0.5, 1.0) or mock control at 3 dpi. Sox2 is a hallmark protein expressed in NPCs. Scale bar, 50 μm. **g** qRT-PCR analyses of ZIKV pre-membrane mRNAs relative to GAPDH extracted from FD, FV, H&S NPCs infected with ZIKV at different MOI (0.25, 0.5, 1.0) or mock control at 2 dpi. Quantification data are presented as mean ± SEM. *n* = 3. **h**–**j** qRT-PCR analyses of regional marker genes Pax6, Nkx2.1 and Hoxb4 mRNAs relative to GAPDH extracted from FD, FV, H&S NPCs infected with ZIKV at 0.5 MOI or mock control at 3 or 6 dpi. Quantification data are presented as mean ± SEM. *n* = 3

We and others established a sophisticated procedure to specify hESCs to primitive neuroectoderm cells (pNE), definitive neuroepithelial cells (dNE), and NPCs with committed regional identities formed organoid structures in the absence or presence of patterning morphogens (Fig. 1a) [16, 25–27]. At days 14–17 of neural differentiation under chemically defined conditions, hESC-derived dNE cells presented typical columnar neuroepithelial cell morphology and organized into neural tube-like rosettes (Fig. 1a). The hESCs uniformly expressed Oct4 and Sox2, hallmark genes of pluripotency (Fig. 1b). At the pNE stage, cells lost Oct4 and began to express Pax6 (Fig. 1c) [25]. Without patterning morphogens, NPCs within the organoid adopted an FD identity, which exclusively expressed FoxG1 and Pax6 (Fig. 1a, d, h–J). Sonic hedgehog (Shh) and retinoid acid (RA) are two potent patterning morphogens that ventralize and caudalize pNE along the dorsoventral and anteroposterior axes, respectively. The yielded FV NPCs patterned through Shh expressed FoxG1 and Nkx2.1, whereas the H&S NPCs patterned through RA expressed HoxB4 and Pax6 (Fig. 1a, d, h–j) [16, 28]. Confocal images further showed that brain organoids at day 23 of FD, FV and H&S regional identities mostly composed of Sox1-expressing NPCs, while the population of differentiated neurons were less than 0.05% as revealed by a small fraction of Map2, Tuj1 and NeuN immune-labeled cells at the periphery of the organoids (Fig. S1A).

It is unclear whether human NPCs with various regional identities are equally vulnerable to ZIKV infection. We then exposed FD, FV and H&S organoids to ZIKV SZ01 from day 17 to day 23 (Fig. 1e). Immunostaining with an antiserum that recognized ZIKV envelope protein (ZIKV E) confirmed abundant viral envelope proteins clustered round the nucleus in all three organoids, especially at multiplicity of infection (MOI) = 0.5 and 1.0 (Fig. 1f). Quantitative analysis showed increased transcription of viral pre-membrane RNA (Fig. 1g), envelope RNA, and NS5 RNA (Data not shown) in the three regional organoids, which plateaued at a MOI  =0.5–1. Western blot showed ZIKV E protein increased with similar patterns to ZIKV RNAs (Fig. S1B, S1C). Infection of three regional organoids with ZIKV did not affect the regional identities of all three NPCs (Fig. 1h–j). Thus, ZIKV showed similar infection sensitivities in human NPCs with all regional identities.

### ZIKV causes growth arrest and programmed cell death in all regional NPCs

**Fig. 2:**
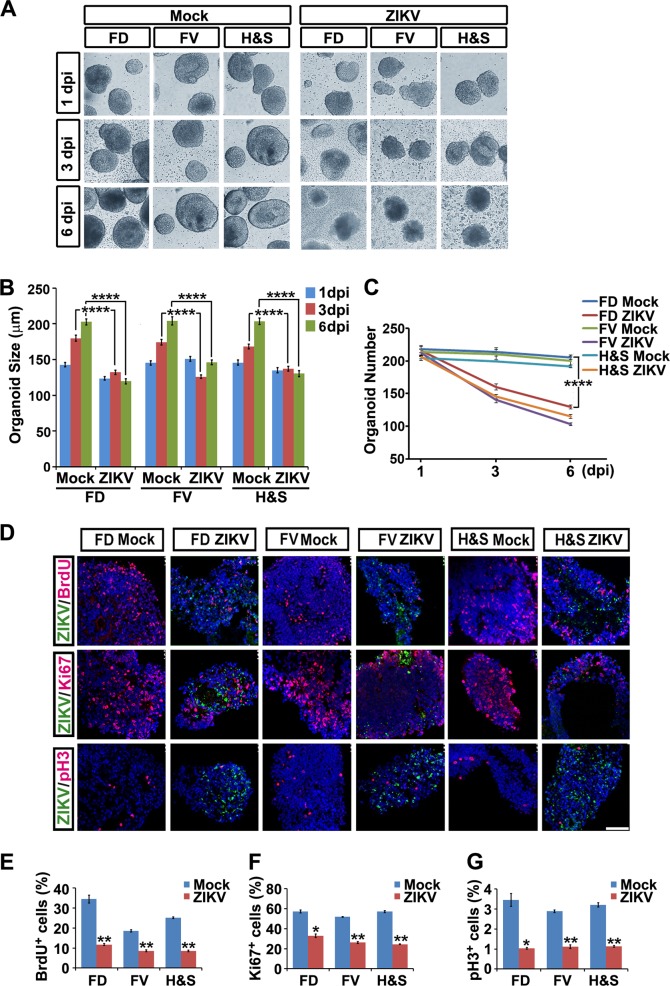
ZIKV abrogates growth of hESC-derived brain organoids of different regional identities. **a** Light images of FD, FV, H&S organoids exposed to mock conditions or ZIKV at MOI =0.5 at 1, 3 or 6 dpi. **b** Size of FD, FV and H&S organoids exposed to mock or ZIKV at MOI = 0.5 at 1, 3 or 6 dpi. Quantification data of organoids diameters are presented as mean ± SEM. *n* = 200 from three independent experiments. Unpaired two-tailed Student’s *t*-test. ****p < 0.0001 compared to mock controls. **c** Numbers of FD, FV and H&S organoids remaining after mock or ZIKV infection at MOI = 0.5 at 1, 3 or 6 dpi. Quantification data are presented as mean ± SEM. *n* = 3. Unpaired two-tailed Student’s *t*-test. ****p < 0.0001 compared to mock controls. **d** Confocal images stained with ZIKV envelope proteins in green, or BrdU, Ki67 and pH3 in red of mock or ZIKV-infected FD, FV, H&S organoids. Scale bar, 100 μm. **e**–**g** Quantitative analyses of percentage of BrdU, Ki67 and pH3 positive cells in mock or ZIKV groups. Data are presented as mean ± SEM. *n* = 3. Unpaired two-tailed Student’s *t*-test. *p < 0.05, **p < 0.01 compared to corresponding mock controls

We photo recorded FD, FV, and H&S organoids at 1, 3, and 6 days post infection (dpi) of ZIKV at an MOI = 0.5. Mock-infected organoids presented expected round and intact shape. Remarkably, ZIKV-infected organoids of all regional identities stopped growing at 3 and 6 dpi (Fig. 2a–c). Two hours of BrdU (50 mM, 1:5000) incorporation analysis and immune-labeling of mitotic markers Ki67 and pH3 all showed significant reduction at 3 dpi in all three regional organoids (Fig. 2d–g). These results indicate that ZIKV infection leads to cell growth arrest and phenocopies microcephaly in all spectra of the regional NPCs.

**Fig. 3:**
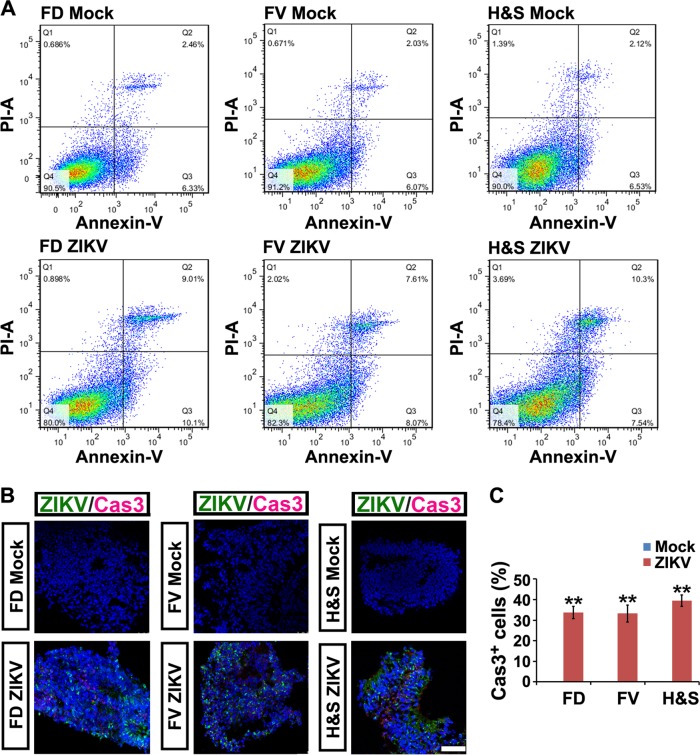
ZIKV induces programmed cell death of hESC-derived NPCs. **a** Annexin V-FITC/PI staining followed by flow cytometry analyses of cell apoptosis in mock and ZIKV groups (MOI=0.006 for 6 days). Q1, dead cells and cell debris. Q2, late apoptotic cells. Q3, early apoptotic cells. Q4, living cells. **b** Confocal images stained with ZIKV envelope proteins in green and cleaved Caspase 3 in red of mock or ZIKV-infected FD, FV and H&S organoids at 3 dpi. Scale bar, 100 μm. **c** Percentage of cleaved Caspase 3 positive cells in mock and ZIKV-infected groups. Data are presented as mean ± SEM. *n* = 3. Unpaired two-tailed Student’s *t*-test. **p<0.01 compared to mock controls

ZIKV infection induced extensive cell death, as observed by the irregular organoid morphology and cell debris detached from the organoids in all three groups (Fig. 2a). The numbers of organoids were also remarkably reduced at 3 and 6 dpi with an MOI = 0.5 (Fig. 2c). Fluorescein isothiocyanate-conjugated annexin V and propidium iodide were used for assessing early and late apoptotic cells with MOI = 0.006 to avoid immediate cell death at higher MOI induced by ZIKV. The percentage of apoptotic NPCs increased 3- to 4-fold at 6 dpi in all three groups (Fig. 3a). This was further confirmed by immunostaining of cleaved caspase 3, a marker of programmed cell death (Fig. 3b, c).

### ZIKV infection strongly induces antiviral immune responses in human NPCs

**Fig. 4:**
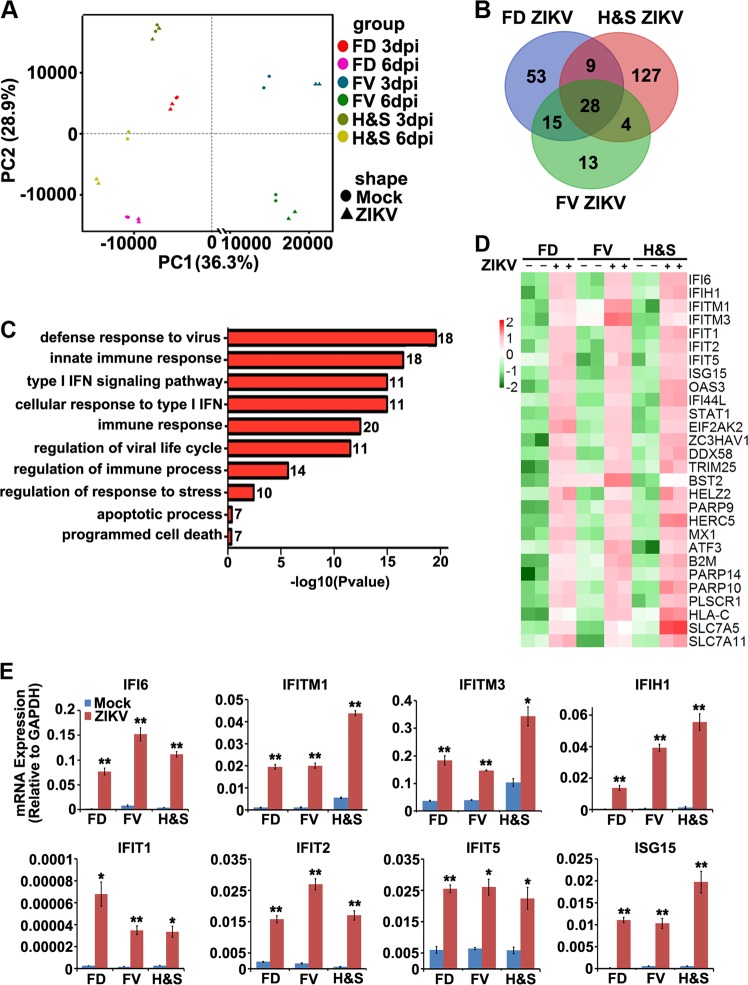
ZIKV infection induces antiviral immune response through activation of ISGs. **a** Principal component analysis of global RNA sequencing from mock (round) and MOI = 0.5 ZIKV-infected (triangle) FD, FV, H&S organoids at 3 dpi or 6 dpi. **b** Upregulated genes in ZIKV-infected organoids at 6 dpi compared to mock groups. 28 genes were significantly upregulated in all three ZIKV-treated regional organoids. **c** List of Gene Ontology (GO) classification of the common 28 upregulated genes and the corresponding number. **d** Heatmaps of 28 upregulated genes in ZIKV-infected FD, FV and H&S organoids. The RPKM values were log2-transformed and row-scaled. **e** qRT-PCR analysis of mRNA expression of 8 ISGs of MOI = 0.5 ZIKV infected vs mock infected FD, FV and H&S organoids at 3 dpi. Data are presented as mean ± SEM. *n* = 3. Unpaired two-tailed Student’s *t*-test. *p < 0.05, **p < 0.01 compared to mock controls

To investigate the cellular pathologies in human NPCs after ZIKV infection, we performed global transcriptome profiling of the FD, FV, and H&S organoids at 3 and 6 dpi. Principal component analysis (PCA) plots showed that samples were primarily clustered by their regional identities and differentiation stages; suggesting ZIKV infection did not significantly affect neural developmental programs. ZIKV infection caused mild, but consistent gene expression changes, which were mostly upregulated and became predominant at 6 dpi (Fig. 4a). We found 105 upregulated genes in the FD NPCs, 60 genes in the FV NPCs, and 168 genes in the H&S NPCs, with at least 1.5-fold differences found between ZIKV-infected groups and the control groups at 6 dpi. Among them, 28 genes overlapped in all three regional NPCs (Fig. 4b). Gene Ontology (GO) analyses of these 28 common genes exclusively referred to the pathways associated with immune responses to viral infection, especially the type I IFN signaling pathway (Fig. 4c). Heatmap analyses further indicated that these 28 genes were strongly upregulated in all three groups (Fig. 4d). While these 28 genes could be clustered to the ISG family [14], we did not identify IFN mRNA induction in all three groups under ZIKV infection (Fig. S2A), nor the type I IFN, indicating the acute antiviral response is mediated by the IFN-independent ISG activation pathway. ZIKV infection-induced ISG activation was also validated by quantitative RT-PCR analyses in all three regional organoids (Fig. 4e). Similar results were observed in a mouse study by infection of ZIKV in the fetal mouse brain at E13.5–16.5 (Fig. S2B and S2C) [4].

### Activation of ISGs through IFNβ administration causes growth arrest in human NPCs

**Fig. 5:**
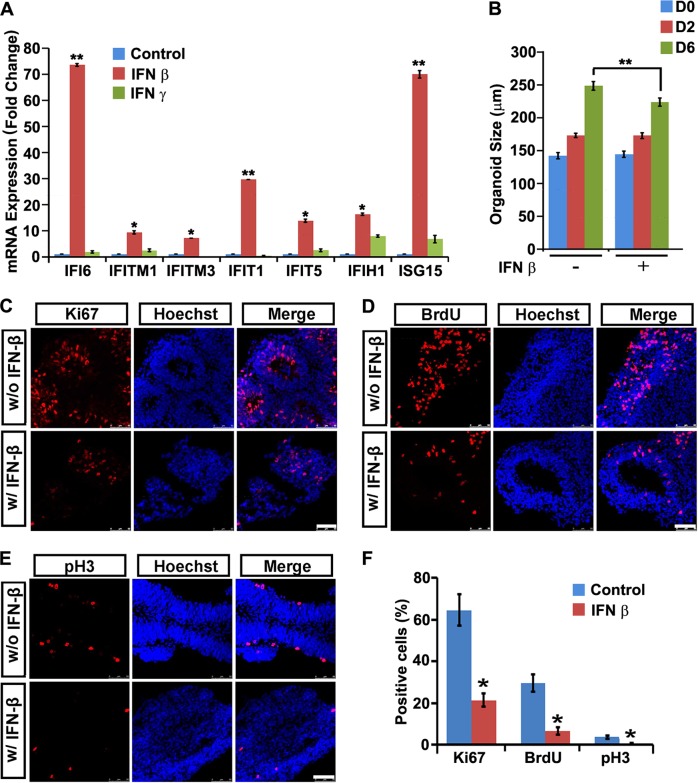
Activation of ISGs by IFNβ administration causes NPC growth arrest. **a** qRT-PCR analysis of ISG mRNA expression of FD organoids treated with 40 ng/ml IFNβ or IFNγ or untreated control for 6 days. Data are presented as mean ± SEM. n=3. Unpaired two-tailed Student’s *t*-test. *p < 0.05, **p < 0.01 compare to untreated controls. **b** Size of FD organoids treated with or without 40 ng/ml IFNβ for 0, 2 and 6 days. Data are presented as mean ± SEM. *n* = 200 from three independent experiments. Unpaired two-tailed Student’s *t*-test. **p < 0.01 compared to the untreated controls. **c**–**e** Confocal images stained with Ki67, BrdU or pH3 in red and Hoechst nuclei staining in blue of untreated or 40 ng/ml IFNβ−treated FD organoids for 3 days. Scale bar, 100 μm. **f** Percentage of Ki67, BrdU and pH3 positive cells. Data are presented as mean ± SEM. *n* = 3. Unpaired two-tailed Student’s *t*-test. *p < 0.05 compared to untreated controls

All three regional NPCs expressed abundant type I and type II IFN receptors (Fig. S2A). The FD, FV, and H&S organoids were then treated with IFNβ or IFNγ for 6 d. Quantitative RT-PCR analyses revealed IFNβ, but not IFNγ administration, induced the expression of those key ISGs efficiently (Fig. 5a, S3A, S3B). Strikingly, IFNβ treatment for 6 d hindered the expansion of the FD, FV, and H&S NPCs, as demonstrated by the reduced organoids diameters (Fig. 5b, S3C). BrdU incorporation studies and immune-labeling of Ki67 and pH3 showed a dramatic reduction in positively labeled cells in all three regional organoids after IFNβ treatment (Fig. 5c–f, S3D, S3E). FACS analysis for apoptotic cells of regional organoids with IFNβ administration showed that the percentage of apoptotic NPCs remained unchanged upon IFNβ administration (Fig. S4A,S4B). Thus, IFNβ could not trigger NPCs apoptosis, but dampen NPCs growth. These data indicate that overactivated ISG pathways might be detrimental for human NPCs of various regional identities.

### Protection of ZIKV infection-induced neuropathy by abrogating ISG induction

**Fig. 6:**
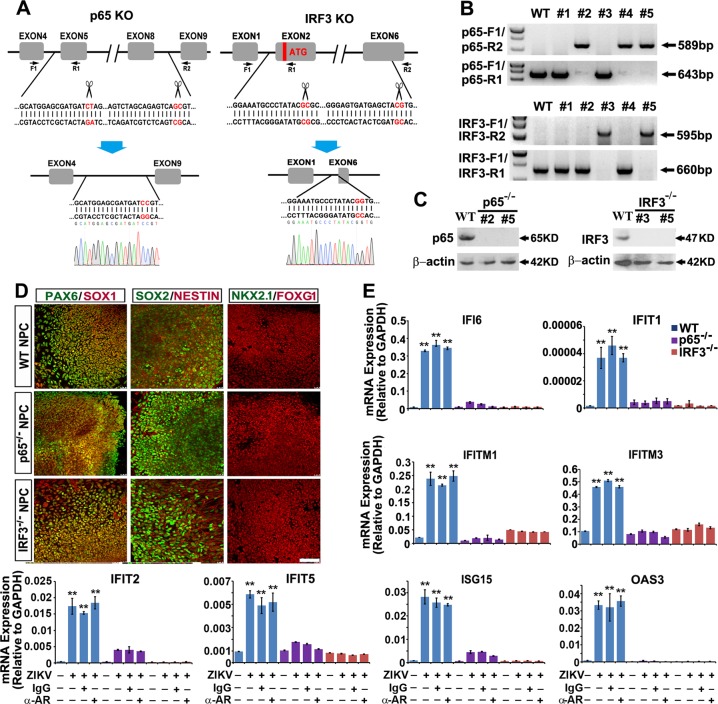
Knockout of p65 or IRF3 abrogates ISG activation upon ZIKV infection. **a** Schematic representation of the paired-KO strategy for NF-κB p65 or IRF3 knockout. The cleavage sites are pointed out by the scissors. Genomic DNA sequences before and after the paired-gRNA mediated cleavage and repair are shown in red. A representative Sanger sequencing peak map verifies precise re-joining of the double blunt ends. The positions of the designed primer sets for genomic PCR are shown as arrows. **b** Genomic DNA PCR results showing 5 colonies retrieved from p65 or IRF3 paired-KO in H9 hESCs. Clones #2, #4 and #5 are p65 KO (upper). Clones #3 and #5 are IRF3 KO (lower). **c** Western blot results further verify complete lack of target protein expression of clones #2, #5 of p65 KO, and #3, #5 of IRF3 KO. **d** Confocal images showing NPCs within FD organoids from WT, p65 KO or IRF3 KO are all Pax6^+^/Sox1^+^/Sox2^+^/Foxg1^+^/Nestin^+^/Nkx2.1^−^. Scale bar, 100 μm. **e** qRT-PCR analysis of ISG mRNA expression of WT, p65 KO and IRF3 KO FD organoids infected with ZIKV at MOI = 0.5 alone or with IFNAR blocking antibody (2 μg/ml, mouse IgG2a, pbi assay science), or control mouse IgG2a (2 μg/ml, mouse IgG2a, R&D) at 5 dpi as well as each mock groups. Data are presented as mean ± SEM. *n* = 3. Unpaired two-tailed Student’s *t*-test. **p < 0.01 compared to mock controls

Infection of RNA viruses or their production of RNA intermediates can activate a subset of ISGs through activation of IRF3, IRF7 and NF-κB [12–14]. NPCs expressed high levels of IRF3 and NF-κB subunit p65 (RELA) [29], and basal level of IRF7 according to our profiling data (data not shown). We therefore ablated p65 and IRF3 in hESCs through donor-free paired gRNA-guided CRISPR/Cas9 knockout (paired-KO) system we established (Fig. 6a) [30]. The KO genotype was verified via genomic DNA PCR analyses (Fig. 6b). Western blotting further confirmed the KO efficacy (Fig. 6c). Ablation of p65 or IRF3 in hESCs did not interfere regular neural differentiation programs and ZIKV infection (Fig. 6d, S1D). Quantitative RT-PCR analyses revealed that ZIKV infection-triggered ISGs expression was almost completely abrogated in p65 or IRF3 KO organoids (Fig. 6e). In all ISGs tested, administration of IFNAR blocking antibody could not ameliorated ZIKV-induced ISGs activation, supporting an IFN-independent ISG induction via activation of IRF3 and NF-κB upon ZIKV infection in NPCs.

**Fig. 7:**
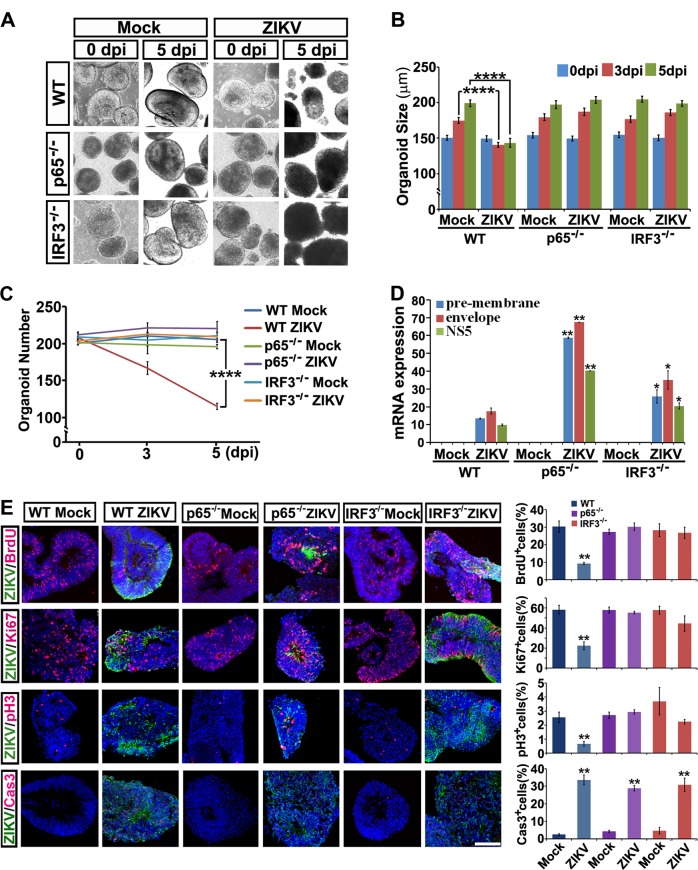
Knockout of p65 or IRF3 in hESCs protects brain organoids from ZIKV infection-induced neuropathy. **a** Light images of FD organoids differentiated from WT, p65 or IRF3 KO hESCs exposed to mock conditions or ZIKV at MOI = 0.5 at 0 or 5 dpi. **b** Size of WT, p65 or IRF3 KO FD organoids exposed to mock or ZIKV at MOI = 0.5 at 0, 3 or 5 dpi. Quantification data are presented as mean ± SEM. n = 200 from three independent experiments. Unpaired two-tailed Student’s *t*-test. ****p < 0.0001 compared to mock controls. **c** Number of WT, p65 or IRF3 KO organoids remaining with mock or ZIKV infection at MOI = 0.5 at 0, 3 or 5 dpi. Quantification data are presented as mean ± SEM. *n* = 3. Unpaired two-tailed Student’s *t*-test. ****p < 0.0001 compared to the mock control. **d** qRT-PCR analyses of ZIKV pre-membrane, envelope and NS5 mRNAs relative to GAPDH extracted from WT, p65 or IRF3 KO organoids infected with ZIKV at 0.5 MOI or mock control at 5 dpi. Quantification data are presented as mean ± SEM. *n* = 3. Unpaired two-tailed Student’s *t*-test. *p < 0.05, **p < 0.01 compared to the corresponding WT controls. **e** Confocal images stained with ZIKV envelope proteins in green, or BrdU, Ki67, pH3 and cleaved Caspase 3 in red of mock or ZIKV-infected WT, p65 or IRF3 KO organoids at 5 dpi. Scale bar, 100 μm (left). Quantitative analyses of percentage of BrdU, Ki67, pH3 and cleaved Caspase 3 positive cells of WT, p65 or IRF3 KO organoids in mock or ZIKV groups (right). Data are presented as mean ± SEM. *n* = 3. Unpaired two-tailed Student’s *t*-test. **p < 0.01 compared to corresponding mock controls

In striking contrast to the wild type (WT) control, ZIKV-infected p65 or IRF3 KO organoids showed similar growth rates as that of their uninfected groups (Fig. 7a–c). Quantitative RT-PCR analyses and Western blot showed significant increase of viral transcripts and envelope protein in p65 or IRF3 KO organoids compared to the WT control, indicating the KO cells are more sustainable for a higher viral burden (Fig. 7d and S1D). Of note, BrdU incorporation studies and immune-labeling of Ki67 and pH3 showed a significant restoration in KO organoids with ZIKV infection (Fig. 7e). These data suggest that ZIKV infection-induced growth arrest of human NPCs could be largely protected by abrogation of the IFN-independent ISG pathway. Interestingly, cleaved caspase 3 staining experiments and FACS analysis showed that the apoptosis of the ZIKV-infected NPCs were not equally protected by ablation of p65 or IRF3 (Fig. 7e, S4C, S4D), possibly because of the severer viral burden in these NPCs lack of ISG activation.

Activation of the innate immune receptor TLR3 leads to nuclear accumulation of NF-κB and IRF3 and subsequently boosts ISGs expression. To investigate the link between ZIKV-mediated overactivation of antiviral responses and TLR3 pathway [31], qRT-PCR analyses were performed to check the basal TLR3 mRNA level of WT, p65 or IRF3 KO organoids, or after stimulation with TLR3 agonist poly (I:C), or combined with TLR3 competitive inhibitor, thiophenecarboxamidopropionate compound. Compared to the positive control of PH5CH8 cells, TLR3 agonist poly(I:C) failed to robustly induce TLR3 mRNA expression in human NPCs (Fig. S5A), nor did ZIKV infection (Fig. S5B). To further test the role of TLR3 pathway in ZIKV-mediated neuropathy, WT, p65 or IRF3 KO organoids were challenged with TLR3 agonist poly (I:C) or combined with TLR3 competitive inhibitor. Activation or blockage of TLR3 signaling for 3 or 6 days showed no obvious effects in the growth rates of human NPCs (Fig. S5C). Moreover, TLR3 competitive inhibitor could not ameliorate ZIKV-mediated neuropathy of NPC organoids (Fig. S5D). Therefore, in our system, ZIKV infection-mediated microcephaly phenotype results from IFN-independent ISG overactivation rather than boosting TLR3 signaling.

## Discussion

The mechanisms underlying ZIKV infection-triggered microcephaly in human newborns remain poorly understood. Our results demonstrated that ZIKV directly invaded and robustly replicated in the brain and spinal cord organoids derived from human pluripotent stem cells. Moreover, ZIKV infection in human NPCs led to cell growth arrest and apoptosis, as evidenced by reduced expression of Ki67 and pH3, lower BrdU incorporation, and increased cleavage of Caspase 3. It has been reported that severe degeneration of the lumbar spinal cord occurs in ZIKV-infected fetuses, which is in line with our conclusion that ZIKV unbiasedly infects all spectra of human FD, FV, and H&S NPCs [20–22, 32, 33].

ZIKV infection during the first trimester of pregnancy causes much severer fetal outcomes than infections occurring in later developmental stages [34–36]. Our results showed that FD, FV, and H&S regional NPCs at day 17 post-initiation of human pluripotent stem cell differentiation were similar to the regional NPCs of 5- to 10-week-old developing embryos [26]. Pathogenesis related to ZIKV infection of in vitro differentiated human NPCs at this stage will thus maximally mirror in vivo neural abnormalities, which model microcephaly in progress. Indeed, transcriptome profiling analyses revealed that ZIKV-infected FD, FV, and H&S regional NPCs had similar molecular signatures on the differentially expressed genes. These results support the effectiveness of in vitro neural differentiation of human pluripotent stem cells in yielding developmentally relevant NPCs for studying neural malformation caused by ZIKV infection.

Type I IFNs (IFN α, β, ε, κ, and ω) elicit a potent antiviral effect in cells by stimulating the IFNα/β receptors (IFNAR1 and IFNAR2) to activate Janus kinase (JAK)-signal transducers and activators of transcription (STAT) signals, leading to the upregulation of hundreds of ISGs for antagonizing viral infection and replication [37, 38]. Type II IFN (IFNγ) is released by immune cells and signals through the IFNγ receptor (IFNGR) to regulate immune responses [39, 40]. Intrauterine inoculation with ZIKV in a mouse model demonstrated that ZIKV infection of the placenta increases production of IFNβ, which, in turn, stimulates ISG expression to limit viral infection [41]. In the brain, astrocytes are also capable of producing IFNs [42]. Interestingly, ZIKV infection in human NPCs failed to induce IFNs. Similar results were observed in the mouse study that ZIKV infection showed under detectable IFN *vs* strongly induced ISGs (Fig. S2). These observations together with ours demonstrate that intracellular processes responsible for viral infection vary significantly among different cell types, which might account for the tissue specific pathology in ZIKV-infected individuals [41, 43–46].

As a key component of innate immune system, TLR3 mediates antiviral response including IFNβ secretion and NFκB signaling activation. By generating cerebral organoids from hESCs, Dang et al. efficiently mimicked microcephaly pathology after ZIKV infection in vitro [31]. In their study, TLR3 was upregulated after ZIKV infection and blocking TLR3 signaling ameliorated the phenotypic effects of ZIKV infection. This is to some extent in line with our current research that ZIKV infection-induced activation of innate immune responses are detrimental for neural cells. However, in our system, TLR3 agonist poly(I:C) and ZIKV infection failed to robustly induce TLR3 mRNA expression (Fig. S5). Moreover, poly(I:C) treatment for 3 or 6 days did not affect normal growth of brain organoids. TLR3 competitive inhibitor also failed to block the phenotypic effects of ZIKV infection. Therefore, in our system, ZIKV infection-mediated microcephaly phenotype results from IFN-independent ISG overactivation rather than boosting TLR3 signaling. We reason that the discrepancy may come from the different neural cell types residing within the brain organoids. We used brain organoids which comprise almost pure NPCs, while in Dang’s study, they used cerebral organoids of a later developmental stage, which are a mixture of NPCs and differentiated neurons. It will be of great interest to investigate whether NPCs, neurons and glial cells have different mechanisms in responding to ZIKV infection.

IRF3 and NFκB transcription factors have strong consensus binding sequences and Chip-seq studies reveal that their binding sites have high co-occurrence. However, IRF3 is more competent than NFκB in recruiting transcriptional machineries to the promoters of target genes, whereas NF-κB is more like an effector for transcription pause release [47]. An open question in our current study is whether IRF3 and NF-κB have equal or differential effects upon ZIKV infection. In our qRT-PCR analyses, the ZIKV pre-membrane, envelope and NS5 mRNAs under the p65 KO are higher than those under IRF3 KO conditions, suggesting a higher viral loads in p65 KO NPCs. Future studies are apparently needed to investigate whether there is a difference of virus entry, virus replication or cell survival between IRF3 and NFκB KO cells upon ZIKV infection.

In a recently published paper, Wu and others showed that stem cells express a subset of ISGs to protect stem cells and their differentiation potential during viral infection [46]. In our current study, ISG activation either by ZIKV infection or IFNβ administration interfered with normal proliferation of human NPCs. Our data thus suggest a detrimental rather than protective role of ISGs, no matter induced via the IFN-dependent or -independent pathways in early human fetal brain upon viral infection. Indeed, among the ISG family, IFIT proteins lead to the accumulation of cells at the G1-S phase transition, or bind and sequester the ribosomal protein to contribute to the antiproliferative capacity [48, 49]. ISG15 has also been reported to inhibit cancer cell growth and promote apoptosis [50].

Taken together, our current study reveals IFN-independent ISG overactivation in human NPCs upon ZIKV infection, and their detrimental role in causing cell growth arrest in human NPCs. We also demonstrate that abrogation of intrinsic ISGs activation might serve a way to mitigate ZIKV infection-induced neuropathy.

## Methods

### Human ESC culture

Human ESC line H9 (WA09, passages 25–45, WiCell Agreement No. 14-W0377) was cultured on a feeder layer of irradiated mouse embryonic fibroblasts (MEFs) as previously described [51]. Cells were passaged every 5 days through dispase (Gibco, 17105) digestion. The components of the human ESC culture medium (hESCM) are: DMEM/F12, 20% knockout serum replacer, 1× MEM non-essential amino acids solution, 1× l-glutamine solution, 0.1 mM β-mercaptoethanol and 4 ng/ml FGF2.

### Construction of the gRNA plasmids

The blank gRNA vector with two BbsI restriction sites was described as before [52]. The gRNA vector was digested with BbsI, gel purified, and ligated to the annealed oligoes containing targeting sequences of designed gRNAs. Sequences of double gRNAs used for NF-κB p65 (RELA) KO were:

p65#1: GCATGGAGCGATGATCTAG,

p65#2: AGTCTAGCAGAGTCAGCGT.

Sequences of double gRNAs used for IRF3 KO were:

IRF3#1: GGAAATGCCCTATACGCGC,

IRF3#2: GGGAGTGATGAGCTACGTG.

Genomic DNA PCR primer sets for validation of NF-κB KO were:

p65 F1: AGTGAAGGGACAGGGTTCGTTG,

p65 R1 AATGGGCACCAAGATTCCAG,

p65 R2: CCAAATCTGCTCCTGTCACCTCT.

Genomic DNA PCR primer sets for validation of IRF3 KO were:

IFR3 F1: TATACTGGCGGAATTGAGGGAG,

IRF3 R1: AAGATTCCGAAATCCTCCTGCTGT,

IRF3 R2: TCCTGAACTTGTCATCTGCCCAC.

### Generation of knockout hESC lines

hESCs were cultured in hESCM with Y-27632 (1 mM, Calbiochem, Y27632), a Rho kinase inhibitor, for 3 h prior to electroporation. Cells were then digested by trypsin for 3 min into single cells and rinsed with PBS twice. 1 × 10^7^ cells were electroporated with appropriate combination of Cas9 plasmids (5 μg) (addgene#44719), two gRNAs (5 μg) and CAG promoter-driven puromycin plasmid (5 μg) in 200 μl of electroporation buffer using the Gene Pulser Xcell System (Bio-Rad) at 250V, 500 μF in a 0.4 cm cuvettes (Phenix Research Products). Cells were treated with puromycin (0.5 μg/ml) from day 2–5 post electroporation. After puromycin selection, MEF-conditioned hESCM was supplied, and drug-resistant colonies could be picked up for genotyping analyses after 10 days post electroporation [30].

### Neural differentiation and ZIKV infection

The procedure for the neural differentiation of human pluripotent stem cells were described previously [51, 53–56]. Briefly, human ESCs were detached from the MEF layers after dispase digestion. ESC aggregates were then pipetted up and down against the bottom of a 50 ml conical centrifuge tube to break up the colonies into 100–200 μm pieces and suspended in hESCM for 4 days to form embryoid bodies (EBs). EBs were then switched to the neural induction medium (NIM) to guide the cells toward the pNE fate for 2 days. At day 6, cell aggregates were plated on laminin-coated culture surface and neural tube-like rosettes could be seen at days 14–17. For regional patterning, Shh (500 ng/ml, 130095730, Miltenyi Biotechnology) or RA (0.1 μM, 302794, Sigma) was added to the pNE cells from day 10–17 to generate FV or H&S regional NPCs, respectively [16, 53]. NPCs were maintained in suspension culture as organoids in NIM. The recipe for NIM is as follows: DMEM/F12, 1× N2 supplement, 1× MEM non-essential amino acids solution and 2 μg/ml heparin. For ZIKV infection, quantified virus were added into the organoid culture for 2 h, and then washed with fresh NIM medium. Culture medium was refreshed every 2 days.

### Virus

ZIKV strain SZ01/2016 (GenBank number: KU866423) was isolated from a patient who returned from Samoa [24]. Virus stocks were prepared by inoculation onto a confluent monolayer of C6/36 mosquito cells. Virus stocks were titrated by plaque forming units (PFU) on BHK-21 cells.

### Plaque assay

ZIKV quantification was completed by plaque assay on BHK-21 cell cultures. Duplicate wells were infected with 0.1 ml aliquots from serial 10-fold dilutions in growth medium and virus was adsorbed for 2 h. Following incubation, the inoculum was removed, and monolayers were overlaid with 3 ml medium containing a 1:1 mixture of 1.2% oxoid agar and 2× DMEM (Gibco, Carlsbad, CA) with 10% (vol/vol) FBS and 2% (vol/vol) penicillin/streptomycin. Cells were incubated at 37 °C in 5% CO_2_ for 4–6 d for plaque development. Cell monolayers then were stained with 2 ml crystal violet formaldehyde solution, and incubated overnight at room temperature before counting the number of plaques.

### mRNA extraction and RT-PCR analysis

Total cellular RNA was isolated using the Trizol kit (Invitrogen) and RNA concentration was determined by NanoDrop 2000c (Thermo Scientific). 1 μg of total RNA from each sample was reverse transcribed into cDNA using SuperScript III (Invitrogen). Real-time PCR was performed (Bio-Rad, CFX Connect Real-Time System) using the Ssofast EvaGreen kit (Bio-Rad). Primer oligonucleotides used for qRT-PCR were as follows:

**Table:** 

Genes	Forward primers	Reverse primers
GAPDH	ATGACATCAAGAAGGTGGTG	CATACCAGGAAATGAGCTTG
IFI6	CTGGTCTGCGATCCTGAATG	AGAGGTTCTGGGAGCTGCTG
IFIM1	ACTCCGTGAAGTCTAGGGACA	TGTCACAGAGCCGAATACCAG
IFIM3	GGTCTTCGCTGGACACCAT	TGTCCCTAGACTTCACGGAGTA
IFIT1	GAAAGCCTCAGTCTTGCAGC	CATCACCATTTGTACAAGAGCCT
IFIT2	AGCGAAGGTGTGCTTTGAGA	GAGGGTCAATGGCGTTCTGA
IFIT5	CGTCCTTCGTTATGCAGCCAAG	CCCTGTAGCAAAGTCCCATCTG
IFIH1	GGGGCATGGAGAATAACTCA	TGCCCATGTTGCTGTTATGT
ISG15	GCCTTCAGCTCTGACACC	CGAACTCATCTTTGCCAGTACA
OAS3	TCTGAGACTCACGTTTCCTGA	CACTGTTGAGGAGGGTAGAGTA
ZIKV prM	CGGAGATCTAGAAGACTGTGAC	ATGACTTTTTGGCTCGTTGAGC
ZIKV E	GATTGAAGGGCGTGTCATACTCC	TCAGTGGAACGGGGTTAGCGG
ZIKV NS5	CTGGTATATGTGGCTAGGGGC	TGGTGATTAAGAGCTTCATTCTCC
TLR3	TTGCCTTGTATCTACTTTTGGGG	TCAACACTGTTATGTTTGTGGGT

### Western blot

Cells were washed three times with PBS and were solubilized in 1×SDS-PAGE loading buffer at 50 ^o^C for 20 min and separated by SDS-PAGE. Primary antibodies used were anti-ZIKV E proteins (1:5000, mouse IgG, Biofront), p65 (1:1000, rabbit IgG, Abcam), IRF3 (1:1000, rabbit IgG, Abcam) and β-actin (1:10,000, Mouse IgG, sigma). Images were developed and semi-quantified by Image J.

### Immunocytochemistry and imaging

Coverslip cultures were fixed in 4% paraformaldehyde for 10 min at room temperature. After adequate washing with PBS, cells were incubated in a blocking buffer (10% donkey serum plus 0.2% Triton X-100 in PBS) for 60 min at room temperature followed by primary antibody incubation at 4 ^o^C overnight. On the next day, coverslips were washed with PBS and stained with the fluorescently conjugated secondary antibodies (1:1000, Jackson, West Grove, PA). Nuclei were counterstained with Hoechst 33258. Coverslips were visualized with Leica TSC SP5 (Leica Microsystems, Bensheim, Germany) confocal laser-scanning microscope. Antibodies used in this study included Pax6 (1:1000, rabbit IgG, Covance), Nkx2.1 (1:400, mouse IgG, Chemicon), FoxG1 (1:1000, rabbit IgG, Abcam), Sox1 (1:500, goat IgG, R&D), Sox2 (1:1000, goat IgG, R&D), Oct4 (1:1000, mouse lgG, Santa Cruz), HoxB4 (1:50, mouse IgG, DSHB), Nestin (1:500, mouse IgG, millipore), Map2 (1:10,000, chicken IgY, abcam), Tuj1 (1:5000, mouse IgG, sigma), cleaved caspase 3 (1:500, rabbit IgG, CST), BrdU (1:200, rat IgG, Abcam), Ki67 (1:500, rabbit IgG, Abcam), pH3 (1:500, rabbit IgG, CST) and ZIKV (1:500, mouse anti-flavivirus group antigen antibody, millipore). Light images were visualized with Leica DMI3000 (Leica Microsystems, Bensheim, Germany) microscope and organoid size was measured with Leica application Suite softwares.

### Flow cytometry analysis

Annexin V-fluorescein isothiocyanate (FITC) and pro-pidium iodide (PI) were used for identification of early apoptotic cells and late apoptotic cells. The organoids were trypsinized into single cells and washed with PBS twice. NPCs were stained by the Annexin V-FITC/PI kit (Beyotime institute of biotechnology, China) for 15 min in the dark. After incubation, the samples were immediately analyzed by FACSCalibur Flow Cytometer (BD Biosciences, San Jose, CA).

### Transcriptome analysis

Three regional organoids were infected with ZIKV at MOI = 0.5 or mock infection, and total RNAs were extracted at 3 dpi or 6 dpi and used for global transcriptome analysis. RNA-seq libraries were generated from duplicated samples per condition using the Next Illumina Ultra RNA library prep kit (NEB) following manufacturer’s protocols. RNA concentration of library was measured using Qubit RNA Assay Kit in Qubit 2.0. The insert size was assessed using the Agilent Bioanalyzer 2100 system (Agilent Technologies, CA, USA) and then accurate quantification was performed with Taqman fluorescence probe of AB Step One Plus Real-Time PCR system and sequenced by an Illumina Hiseq 2500 platform. RNA-seq reads were aligned using tophat v2.1.1. Significantly differentially expressed genes were identified using DESeq2 by comparing reads per kilobase of transcript per million mapped reads (RPKMs) between all pairs of samples with *p* value <0.05 and log2 Fold change >1.5. Principle component analysis (PCA) is performed with R package ggpolot. Gene Ontology analyses on biological process were performed by The Database for Annotation, Visualization and Integrated Discovery (DAVID) v6.8. The gene expression profiles of different samples were displayed as heatmap, the RPKM values were log2-transformed and row-scaled for better visualization. The raw data were deposited in the NCBI’s Sequence Read Archive (accession number, GSE129180).

### Statistical analysis

Data were analyzed using Student’s *t*-test for comparison of independent means with pooled estimates of common variances.

## Supplementary information

Figure S1

Figure S2

Figure S3

Figure S4

Figure S5

Supplementary figure legends
