# Supplementary figures and images for: Protection of ZIKV infection-induced neuropathy by abrogation of acute antiviral response in human neural progenitors
Source: Cell Death Differ. 2019 Apr 5;26(12):2607–21. doi: 10.1038/s41418-019-0324-7 (PMC7224299; doi:10.1038/s41418-019-0324-7)

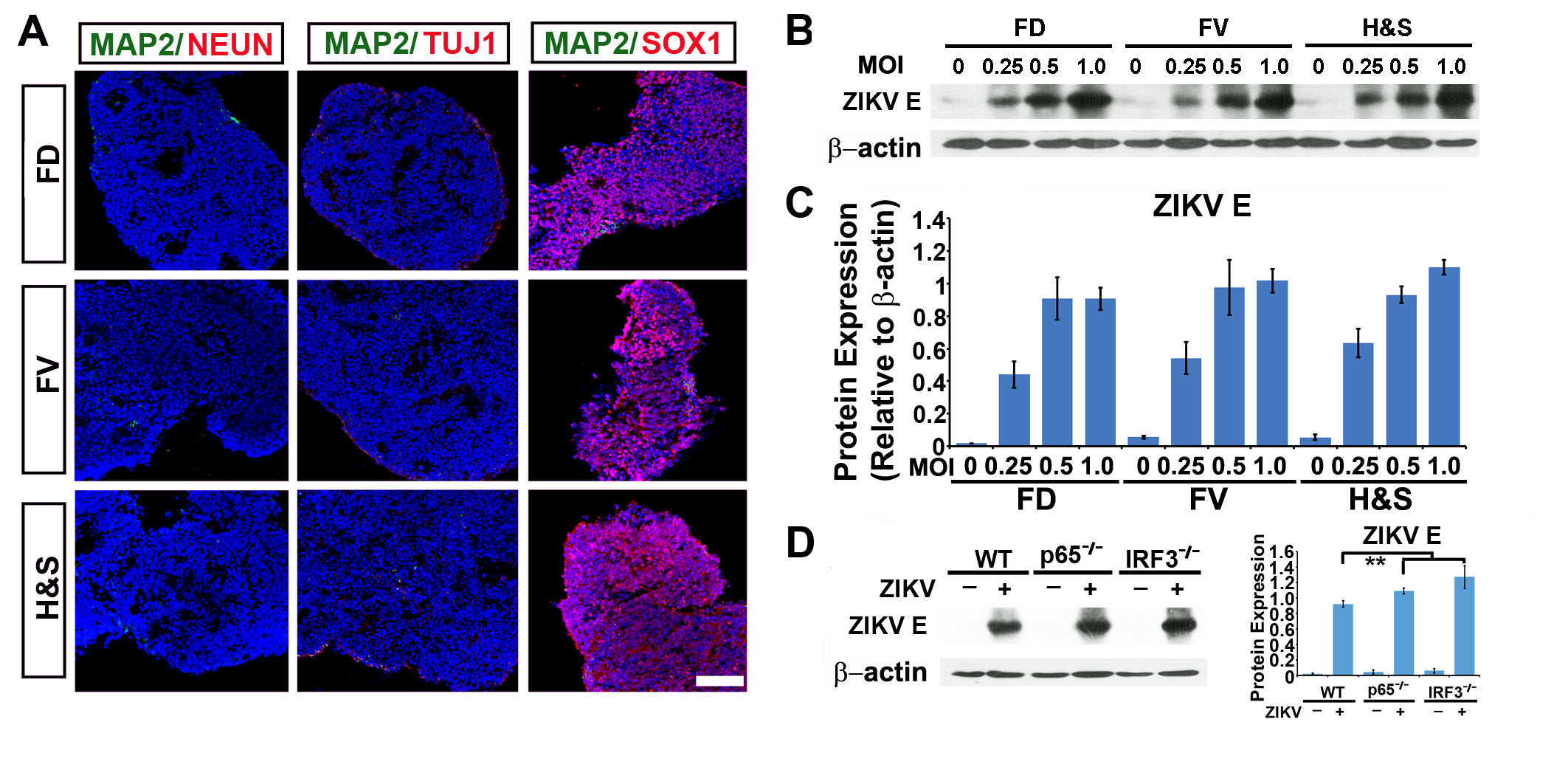

Supplement: Supplementary file 1 — Figure S1 [file 41418_2019_324_MOESM1_ESM.tif]

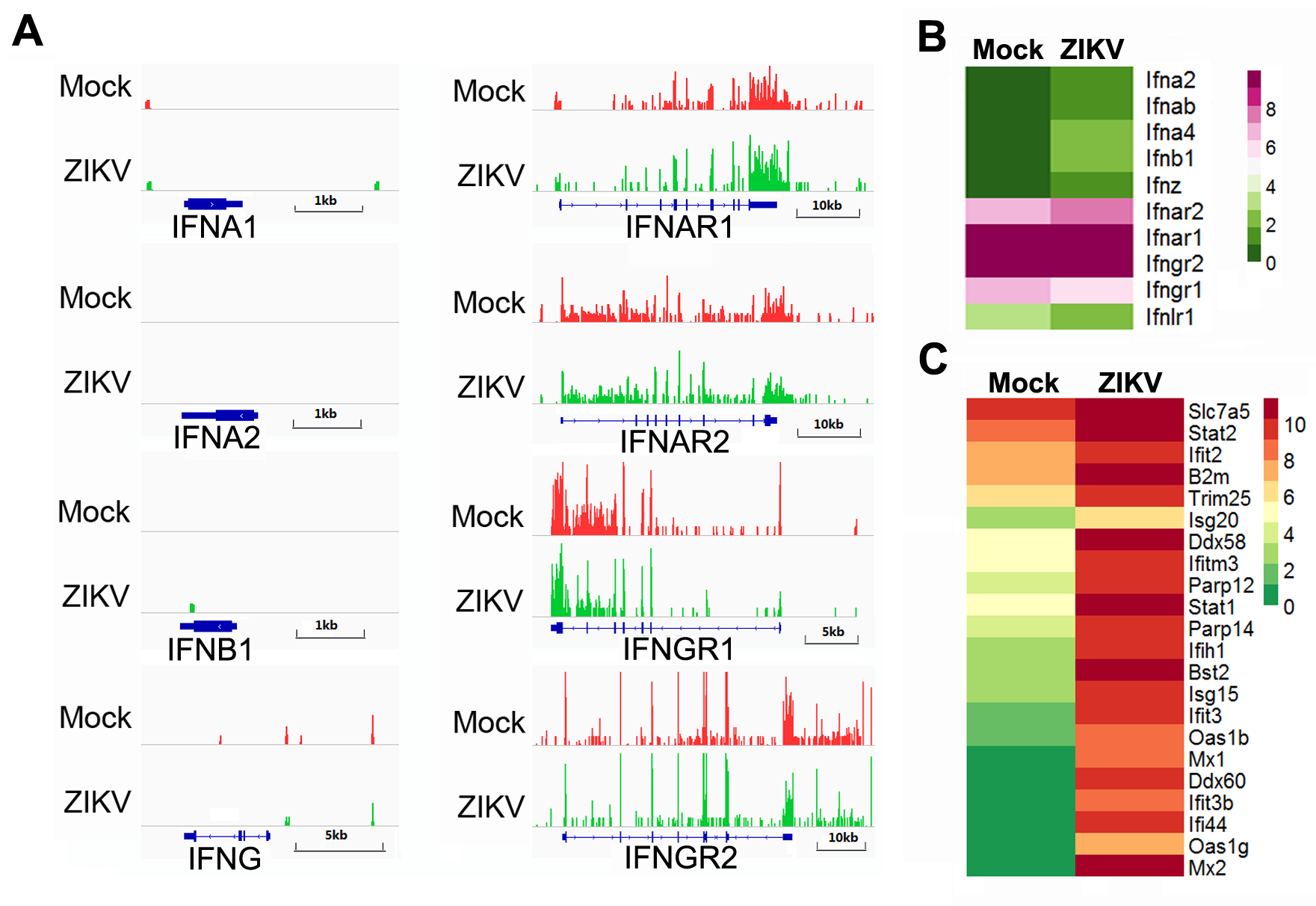

Supplement: Supplementary file 2 — Figure S2 [file 41418_2019_324_MOESM2_ESM.tif]

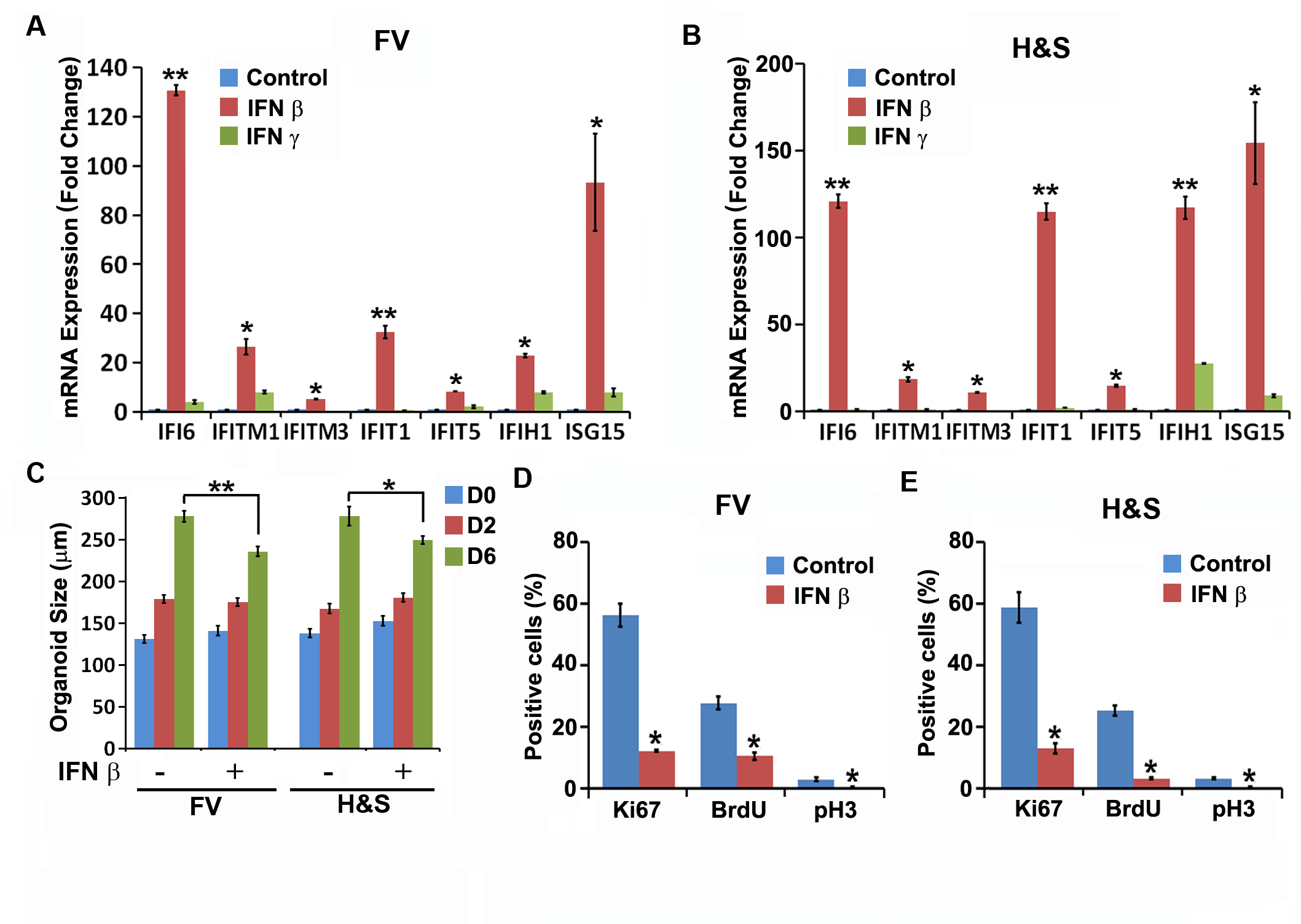

Supplement: Supplementary file 3 — Figure S3 [file 41418_2019_324_MOESM3_ESM.tif]

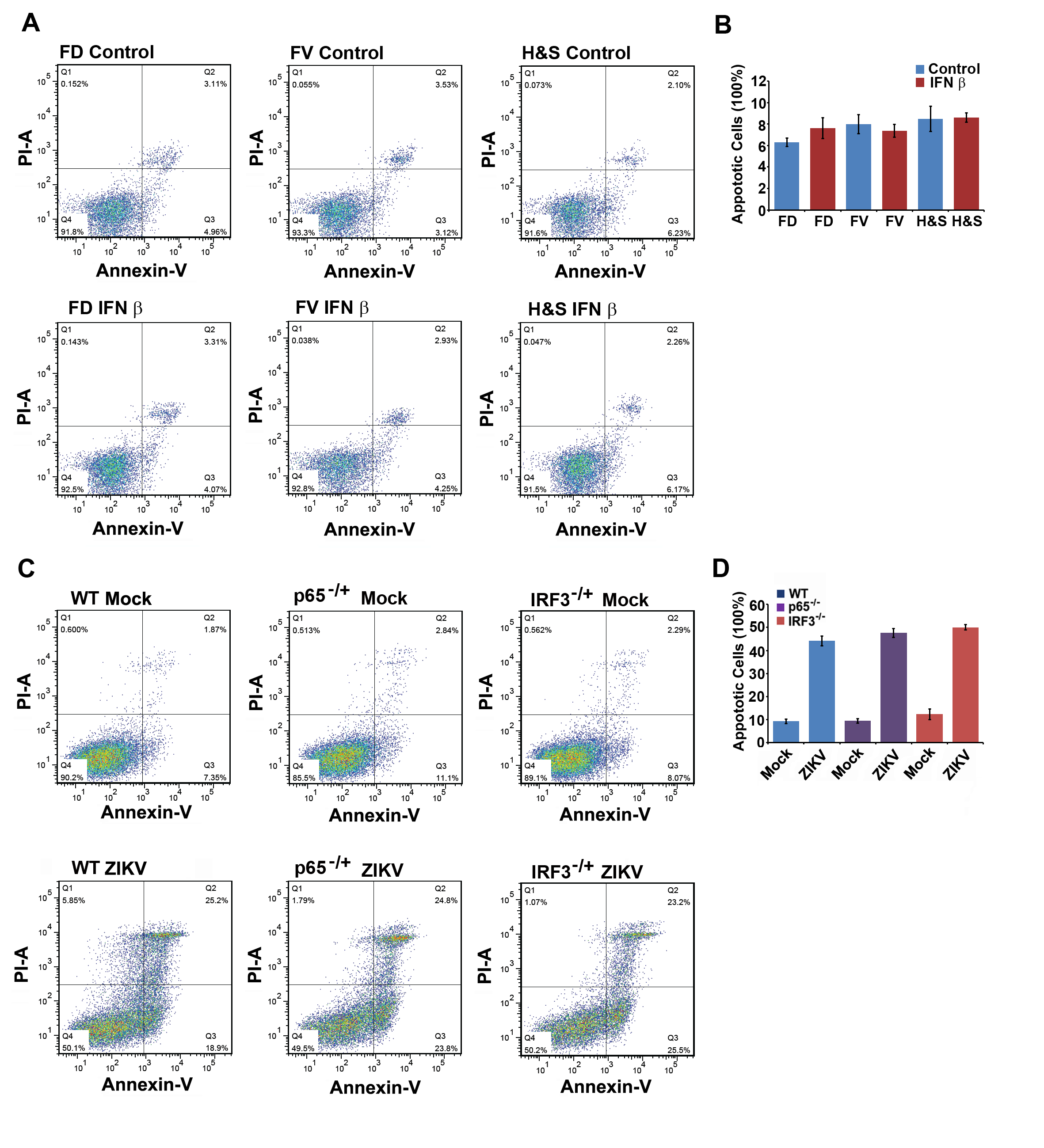

Supplement: Supplementary file 4 — Figure S4 [file 41418_2019_324_MOESM4_ESM.tif]

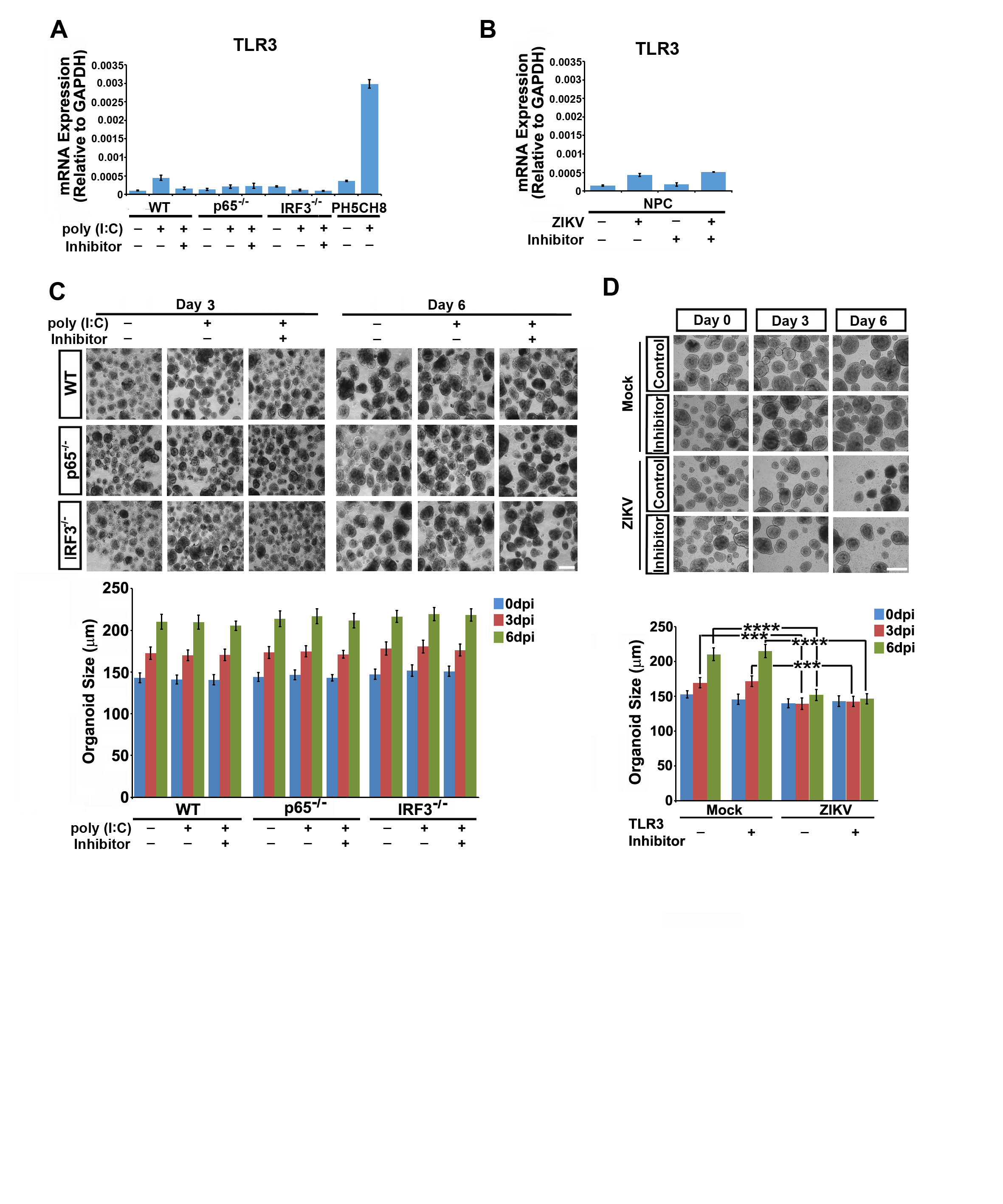

Supplement: Supplementary file 5 — Figure S5 [file 41418_2019_324_MOESM5_ESM.tif]
