# Supplementary material for: Protection of ZIKV infection-induced neuropathy by abrogation of acute antiviral response in human neural progenitors
Source: Cell Death Differ. 2019 Apr 5;26(12):2607–21. doi: 10.1038/s41418-019-0324-7 (PMC7224299; doi:10.1038/s41418-019-0324-7)
Supplement: Supplementary file 6 — Supplementary figure legends [file 41418_2019_324_MOESM6_ESM.docx]

**Protection of ZIKV infection-induced neuropathy by abrogation of acute antiviral response in human neural progenitors**

**Supplementary Figure Legends**

**Figure S1. NPCs in regional brain organoids robustly express ZIKV protein upon ZIKV infection.**

(**A**) FD, FV and H&S organoids were plated onto coverslips for immunostaining. All three regional organoids composed of Sox1 expressing NPCs, but rarely had Map2, Tuj1, or NeuN expressing neurons. Scale bar, 100 μm.

(**B**, **C**) Western blot of ZIKV envelope protein from FD, FV, H&S NPCs infected with ZIKV at different MOI (0.25, 0.5, 1.0) or mock control at 2 dpi. β-actin was served as an internal control. Semi-quantification data are presented as mean +/- SEM. n=3.

(**D**) Western blot of ZIKV envelope protein from WT, p65 or IRF3 KO organoids infected with ZIKV at MOI=0.5 or mock control at 2 dpi. β-actin was served as an internal control. Semi-quantification data are presented as mean +/- SEM. n=3. Unpaired two-tailed Student’s *t*-test. ** p<0.01 compared to corresponding WT control infected with ZIKV.

**Figure S2. ZIKV infection induces robust ISG activation, which is independent of IFN expression.**

(**A**) Left panel, IGV Browser Views of type I IFNs including IFNA1, IFNA2 and IFNB1 and type II IFN, IFNG, in mock or ZIKV treated FD organoids. Right panel, IGV Browser Views of the expression of type I IFN receptors including IFNAR1 and IFNAR2 as well as type II IFN receptors, IFNGR1 and IFNGR2, in mock or ZIKV treated FD organoids.

(**B**, **C**) Heatmaps of IFNs, IFN receptors (B) and ISGs (C) from Li’s RNA-seq data of ZIKV-infected and mock-infected littermate brains (E13.5-E16.5) ^[4]^. The RPKM values were log2-transformed and row-scaled.

**Figure S3.** **IFNβ administration triggered ISG activation inhibits proliferation of FV and H&S NPCs.**

(**A**, **B**) qRT-PCR analyses of ISG mRNA expression of FV (A) and H&S (B) organoids treated with 40 ng/ml IFNβ or IFNγ or untreated for 6 days. Data are presented as mean +/- SEM. n=3. Unpaired two-tailed Student’s *t*-test. * p<0.05, ** p<0.01 compared to the untreated controls.

(**C**) Size of FV and H&S organoids treated with or without 40 ng/ml IFNβ for 0, 2 and 6 days. Data are presented as mean +/- SEM. n=200. Unpaired two-tailed Student’s *t*-test. * p<0.05, ** p<0.01 compared to the untreated controls.

(**D**, **E**) Percentage of Ki67, BrdU and pH3 positive cells in FV (D) and H&S (E) organoids treated with or without 40 ng/ml IFNβ for 6 days. Data are presented as mean +/- SEM. n=300. Unpaired two-tailed Student’s *t*-test. * p<0.05 compared to the untreated controls.

**Figure S4.** **ZIKV but not INF β induces programmed cell death of hESC-derived NPCs, which could not be rescued by knockout of p65 or IRF3.**

(**A, B**) FD, FV or H&S organoids were treated with or without 40 ng/ml IFN β for 6 days. Annexin V-FITC/PI staining followed by FACS analyses of cell apoptosis in control and IFN β groups. Q1, dead cells and cell debris. Q2, late apoptotic cells. Q3, early apoptotic cells. Q4, living cells. Quantification data are presented as mean +/- SEM. n=3.

(**C, D**) WT, p65 or IRF3 KO organoids exposed to mock or ZIKV at MOI=0.5 at 6 dpi. Annexin V-FITC/PI staining followed by FACS analyses of cell apoptosis in mock and ZIKV infected groups. Quantification data are presented as mean +/- SEM. n=3.

**Figure S5.** **ZIKV-mediated NPC growth arrest is TLR3-independent.**

(**A**) qRT-PCR analyses of TLR3 mRNA expression of WT, p65 or IRF3 KO organoids stimulated with TLR3 agonist poly (I:C) (sigma P9582) at 1μg/ml or combined with TLR3 competitive inhibitor at 10 μM for 3 days. TLR3 mRNA expression of PH5CH8 cells challenged with poly (I:C) at 1μg/ml for 8 hrs was served as a positive control. Quantification data are presented as mean +/- SEM. n=3.

(**B**) qRT-PCR analyses of TLR3 mRNA expression of organoids infected with ZIKV at MOI=0.5 in the presence or absence of TLR3 inhibitor. Quantification data are presented as mean +/- SEM. n=3.

(**C**) Light images of WT, p65 or IRF3 KO organoids stimulated with TLR3 agonist poly (I:C) or combined with TLR3 inhibitor for 3 or 6 days. Scale bar, 200 μm (upper). Quantification data are presented as mean +/- SEM. n=100 (lower).

(**D**) Light images of organoids infected with ZIKV at MOI=0.5 in the presence or absence of TLR3 inhibitor for 0, 3 or 6 days. Scale bar, 200 μm (upper). Quantification data are presented as mean +/- SEM. n=100. Unpaired two-tailed Student’s *t*-test. *** p<0.001, **** p<0.0001 compared to corresponding mock controls (lower).
